# Phosphodiesterase (PDE) III inhibitor, Cilostazol, improved memory impairment in aluminum chloride-treated rats: modulation of cAMP/CREB pathway

**DOI:** 10.1007/s10787-022-01010-1

**Published:** 2022-06-21

**Authors:** Mona Khalifa, Rania M. Abdelsalam, Marwa M. Safar, Hala F. Zaki

**Affiliations:** 1 Department of Clinical Pharmacy, National Cancer Institute, Cairo University, Qasr Al-Eyni Street, Fom El Khalig, Cairo, 11796 Egypt; 2 Department of Pharmacology and Toxicology, Faculty of Pharmacy, Cairo University, Qasr Al-Eyni Street, Cairo, 11562 Egypt; 3 Department of Pharmacology and Biochemistry, Faculty of Pharmacy, The British University in Egypt, Suez Desert Road, P.O. Box 43, El Sherouk City, 11837 Cairo Egypt; 4 Department of Biology, School of Pharmacy, Newgiza University, First 6th of October, Giza, 3296121 Egypt

**Keywords:** Cilostazol, Alzheimer’s disease, Aluminum, cAMP/CREB pathway, Tumor necrosis factor-alpha, Neprilysin

## Abstract

The most prevalent type of dementia is Alzheimer's disease (AD), which is currently incurable. Existing treatments for Alzheimer's disease, such as acetylcholinesterase inhibitors, are only effective for symptom relief. Disease-modifying medications for Alzheimer's disease are desperately required, given the enormous burdens that the disease places on individuals and communities. Phosphodiesterase (PDE) inhibitors are gaining a lot of attention in the research community because of their potential in treating age-related cognitive decline. Cilostazol is a selective PDE III inhibitor used as antiplatelet agent through cAMP response element-binding (CREB) protein phosphorylation pathway (cAMP/CREB). The neuroprotective effect of cilostazol in AD-like cognitive decline in rats was investigated in this study. After 2 months of intraperitoneal administration of 10 mg/kg aluminum chloride, Morris water maze and Y-maze (behavioral tests) were performed. After that, histological and biochemical examinations of the hippocampal region were carried out. Aluminum chloride-treated rats showed histological, biochemical, and behavioral changes similar to Alzheimer's disease. Cilostazol improved rats' behavioral and histological conditions, raised neprilysin level while reduced levels of amyloid-beta protein and phosphorylated tau protein. It also decreased the hippocampal levels of tumor necrosis factor-alpha, nuclear factor-kappa B, FAS ligand, acetylcholinesterase content, and malondialdehyde. These outcomes demonstrate the protective activity of cilostazol versus aluminum-induced memory impairment.

## Introduction

Dementia is a major health issue, being correlated with significant mortality and morbidity (Ballard et al. 2011). Dementia affects the elderly in the overwhelming majority of instances, producing age the most significant risk factor. Dementias are categorized depending upon their underlying pathologies, which are largely determined by misfolded proteins aggregate accumulation in neurons and glia, and also in the extracellular matrix, in vulnerable brain regions (Seeley et al. 2009).

The buildup of abnormally folded protein fragments, including amyloid-beta (Aβ) and tau proteins, which form amyloid plaques (Aβ plaques) and neurofibrillary tangles (NFTs), consecutively, characterizes Alzheimer's disease (AD) dementia (Pluta et al. 2013; Singh et al. 2013). Neprilysin (NEP) was first recognized as the major Aβ degrading enzyme utilizing biochemical approaches (Iwata et al. 2000). The NEP gene's deletion or NEP activity inhibition was demonstrated to elevate Aβ levels in AD's mouse models (Eckman et al. 2006; Farris et al. 2007).

Numerous examinations initially indicated that in addition to Aβ plaques and NFTs, the brains of patients with AD appeared proof of a long-term inflammatory reaction (Tuppo and Arias 2005; Mrak and Griffin 2007). Inflammation in the brain appears to get dual function, acting as a neuroprotective factor throughout an acute response but becoming harmful as a chronic response develops (Kim and Joh 2006). Drugs presently used to treat AD have restricted advantages, so there is a necessity for a reliable treatments that will not only supply symptomatic relief but also slow the progression of the disease.

Phosphodiesterase inhibitors as cilostazol were reported to improve cyclic guanosine monophosphate (cGMP) and/or cyclic adenosine monophosphate (cAMP) signaling by reducing these cyclic nucleotides’ degradation (Heckman et al. 2015). As both cGMP and cAMP signaling are crucial to various cellular functions, involving neuroprotection and neuroplasticity, a clinical application of cilostazol for AD is expected (Saito et al. 2016).

As a selective inhibitor of cAMP phosphodiesterase type III, cilostazol elevated levels of cAMP stimulate protein kinase A, ending in platelet aggregation inhibition (Gresele et al. 2011). It also has several pharmacological actions, such as anti-oxidative, anti-apoptotic, and anti-inflammatory impacts in the brain (Hong et al. 2006). It is also recognized to decrease Aβ accumulation and to enhance brain function in an experimental model of AD (Park et al. 2011), and therefore, it appeared interesting to study the possible protective impacts of cilostazol on aluminum-induced memory impairment in rats.

## Material and Methods

### Animals

Male adult Wistar rats weighting between 130 and 150 g were used. Animals were maintained at stable surroundings: 12:12 h light/dark cycle, humidity (60 ± 10%), and temperature (23 ± 1 °C). Before the beginning of the experiments, an adaptation period of 1 week was given for rats to acclimatize with the new conditions, and they were fed rat food and water ad libitum*.*

### Chemicals and drugs

Cilostazol (Pletal®) was bought from Otsuka Pharmaceutical Co., Cairo, Egypt, and aluminum chloride (ALCL_3_) was bought from Sigma-Aldrich, St. Louis, MD, USA. Chemicals used in the present work were of highly pure and of excellent analytical grade.

### Experimental design and treatment protocol

**Fig. 1:**
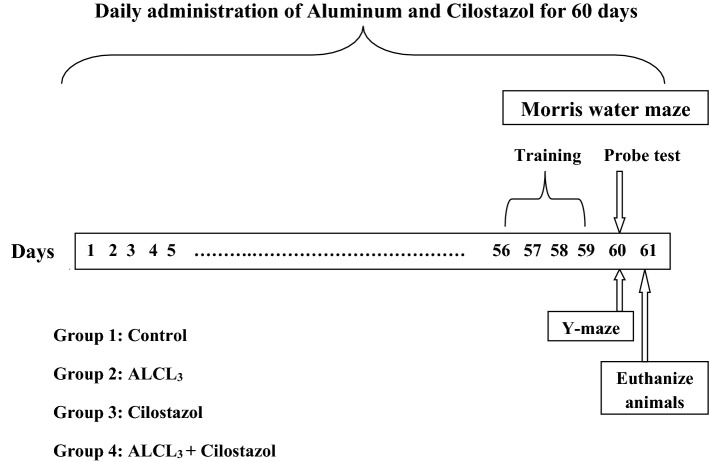
Graphical illustration of the experimental design. ALCL3, aluminum chloride

As described in Fig. 1, four groups of animals (10 rats per group) were required, Group 1 (Control group): rats were given saline, ip, once per day for 60 days. Group 2 (ALCL_3_ group): rats were given ALCL_3_ (10 mg/kg, ip) once per day for 60 days. Group 3 (cilostazol group): rats were given cilostazol (50 mg /kg, po) once per day for 60 days. Group 4 (ALCL_3_ + cilostazol group): rats were given ALCL_3_ (10 mg/kg, ip) and cilostazol (50 mg /kg, po) once per day for 60 days. Four days before the end of the experiment (day 56), rats were trained on Morris water maze test for 4 consecutive days. At the end of the experiment (day 60), Morris water maze probe test was done together with Y-maze spontaneous alternation test.

### Behavioral tests

#### Morris water maze test

Morris water maze test was used to assess spatial learning and memory. The maze consists of rounded container of 60 cm height and 150 cm diameter, and contains water to a depth of 40 cm of temperature 27 ± 1 °C. The container is splitted into four partitions (quadrants) and a portable Plexiglass stand of 8 cm in diameter located inside a specified quadrant. For 4 consecutive days, rats were trained in the maze twice daily for 120 s, where they were allowed to find the stand in the specific quadrant by their own in every time, once the rat finds the stand it was permitted to stay on it for 10 s, however, if it failed to find the stand within this 120 s, it was permitted to stay on it for 30 s. The probe test was done on the fifth day, we removed the stand and each animal was allowed to swim freely in the container for 120 s, during the 120 s, we calculate the index of retrieval which is the time each rat stayed in the target quadrant (i.e., the quadrant in which the stand was there) (Morris 1984; Kim et al. 2012).

#### Y-maze spontaneous alternation test

Y-maze test was done as reported previously (Wall and Messier 2002). The maze consists of three identical arms (A, B, and C), there are equal angles in between them, each arm is 35 cm in height, 40 cm in length, and 12 cm in width. At the end of one of the three arms, we put animals, and then, they were allowed to move freely in the maze for five minutes. To get rid of any residual odors, we cleaned arms well between each animal. When a rat puts his hind paws totally inside one of the three arms, so this was counted as a complete arm entry. An alternation was defined as a triad that contains the three letters (ABC, CAB, etc.). Spontaneous alternation percentage (SAP) was obtained from both total arm entries and number of alternations using the following equation:

$${\text{SAP}}\left( \% \right) = \left( {\left( {\text{number of alternations}} \right)/\left( {{\text{total arm entries}} - {2}} \right)} \right) \times {1}00.$$

### Tissue sampling

Tissue sampling was done on day 61 of the experiment (24 h following behavioral experiments). Under anesthesia, animals were euthanized by decapitation, and brains were then separated quickly to collect hippocampi which were freezed at − 80 °C. For biochemical analysis, the collected hippocampi were homogenized (10% w/v) in phosphate buffer (pH 7.4) to obtain the homogenate. In addition, from each group, two rats were selected randomly to obtain their whole brains, which were placed in formalin (10%) for histopathological assessment.

### Biochemical analysis

Hippocampal contents of neprilysin, phosphorylated tau (P-tau), amyloid-beta 1–42 (Aβ_1−42_), FAS ligand (FAS-L), tumor necrosis factor-alpha (TNF-α), nuclear factor-kappa B (NF-κB), malondialdehyde (MDA), and acetylcholinesterase content (AChE) were detected using the ELISA technique.

### Histopathological examination

Rats’ whole brains were placed in formalin (10%) for 24 h. Brains were dehydrated and washed by alcohol, then cleared in xylene, and finally fixed in paraffin in hot air oven at 56 °C for 24 h. Brain sections (thickness: 3-μm) were stained using hematoxylin and eosin to be ready for examination under a light microscope provided with a camera. Samples histopathological examination and handling were performed by a specialist who did not know samples nature to avoid any bias.

### Statistical analysis

One-way analysis of variance (ANOVA) were used to compare between different groups, and for multiple comparisons, Tukey Kramer’s test was used. GraphPad Prism 6.0 (GraphPad Software, San Diego, CA, USA) was used for statistical analysis. Values were displayed as mean ± SEM, at *p* < 0.05 as the minimum level of significance.

## Results

### Behavioral analysis

#### Morris water maze test

**Fig. 2:**
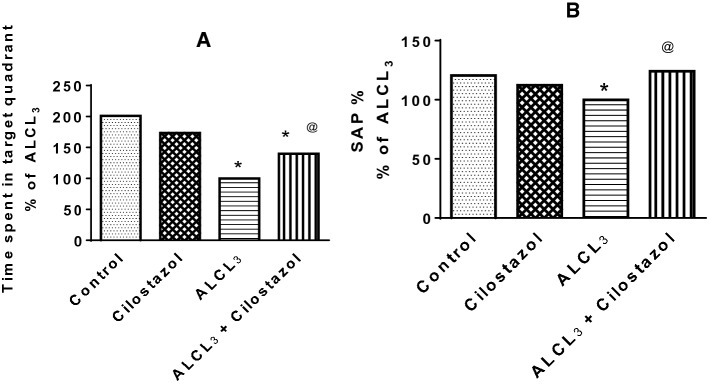
Effect of cilostazol on behavior of rats in Morris water maze (**a**) and Y-maze (**b**) tests in control and ALCL_3_-treated rats. Values are displayed as mean ± SEM (*n* = 10). *Significantly different from control group (*p* < 0.05), ^@^significantly different from ALCL_3_ group (*p* < 0.05)

Time spent by rats treated with aluminum chloride (ALCL_3_) in target quadrant was less than that in control rats. Cilostazol administration to normal rats did not change their behavior in Morris test when compared to control group, while its administration to ALCL_3_-treated rats increased time spent by rats in target quadrant to 139.82% of that in ALCL_3_ group (Fig. 2a).

#### Y-maze spontaneous alternation test

Spontaneous alternation percentage (SAP) decreased significantly in ALCL_3_ group when compared to that in control group. Cilostazol administration to normal rats made no significant difference in SAP in comparison to that in control group. Cilostazol increased SAP when given to ALCL_3_-treated rats when compared to ALCL_3_ group (Fig. 2b).

### Biochemical analysis

#### Effect of cilostazol on hippocampal content of amyloid beta1–42 (Aβ_1−42_), phosphorylated tau (p-tau), and neprilysin in control and ALCL_3_-treated rats

**Fig. 3:**
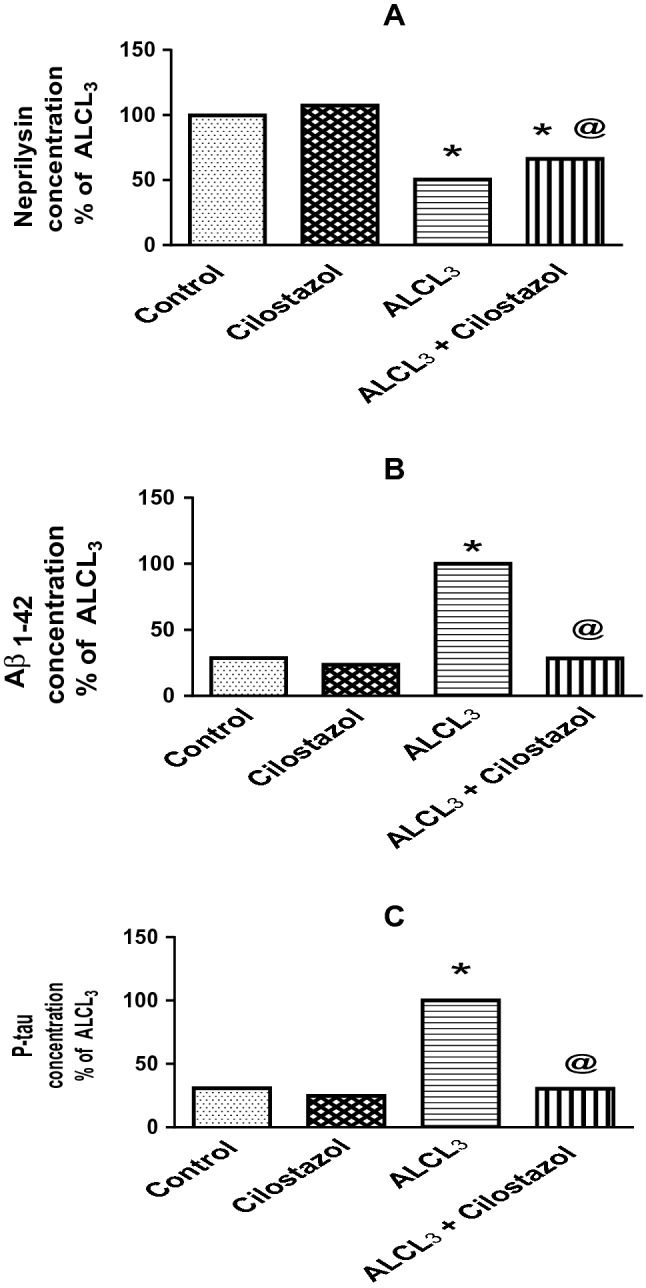
Effect of cilostazol on hippocampal content of neprilysin (**a**), amyloid beta1-42 (Aβ_1–42_, **b**), and phosphorylated tau (p-tau, **c**) in control and ALCL_3_-treated rats. Values are displayed as mean ± SEM (*n* = 10). *Significantly different from control group (p < 0.05), ^@^significantly different from ALCL_3_ group (*p* < 0.05)

Aluminum chloride (ALCL_3_) decreased hippocampal content of neprilysin to 50% (Fig. 3a) and raised hippocampal contents of both Aβ_1–42_ by 3.5-fold (Fig. 3b) and p-tau by 3.2-fold (Fig. 3c) when compared to control group. Cilostazol alone showed no effect on levels of neprilysin, Aβ_1–42_ and p-tau in comparison with control rats, while its administration to ALCL_3_-treated rats increased neprilysin content (1.3-fold) and decreased levels of Aβ_1–42_ (28%) and p-tau (30%) of that in ALCL_3_ group.

#### Effect of cilostazol on hippocampal content of nuclear factor-kappa B (NF-κB), tumor necrosis factor-alpha (TNF-α), and FAS ligand (FAS-L) in control and ALCL_3_-treated rats

**Fig. 4:**
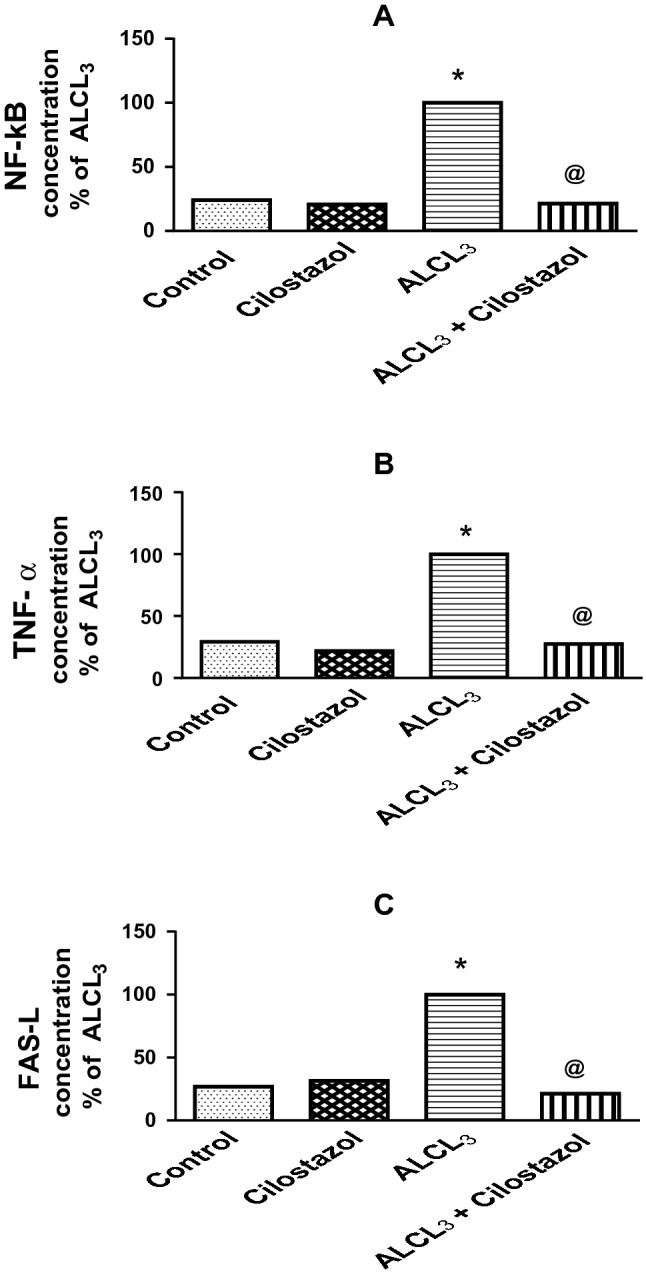
Effect of cilostazol on hippocampal content of nuclear factor-kappa B (NF-κB, **a**), tumor necrosis factor-alpha (TNF-α, **b**), and FAS ligand (FAS-L, **c**) in control and ALCL_3_-treated rats. Values are displayed as mean ± SEM (*n* = 10). *Significantly different from control group (*p* < 0.05), ^@^ significantly different from ALCL_3_ group (*p* < 0.05)

Administration of ALCL_3_ for 60 days significantly increased levels of NF-κB by 4.2-fold (Fig. 4a), TNF-α by 3.4-fold (Fig. 4b), and FAS-L by 3.7-fold (Fig. 4c) in comparison to control group. Cilostazol administered to normal rats showed no change in the previous parameters, while when administered to rats treated with ALCL_3_, it significantly decreased them, NF-κB (21%), TNF-α (27%), and FAS-L (21%) of that in ALCL_3_ group.

#### Effect of cilostazol on hippocampal content of malondialdehyde (MDA) in control and ALCL_3_-treated rats

**Fig. 5:**
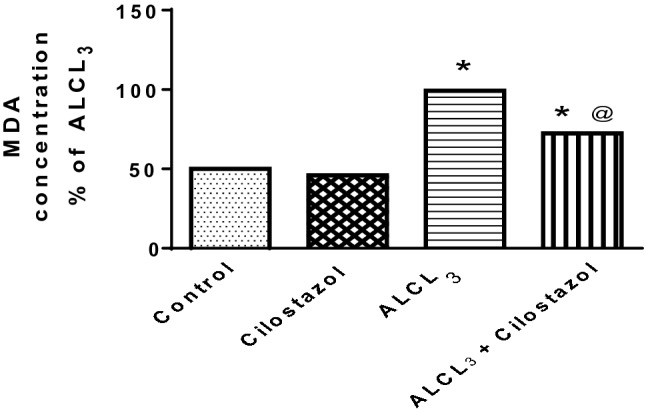
Effect of cilostazol on hippocampal content of malondialdehyde (MDA) in control and ALCL_3_-treated rats. Values are displayed as mean ± SEM (*n* = 10). *Significantly different from control group (*p* < 0.05), ^@^significantly different from ALCL_3_ group (*p* < 0.05)

Aluminum chloride administration raised hippocampal content of MDA by 1.9-fold (Fig. 5). Cilostazol had no effect on MDA level when administered alone, but when given to ALCL_3_-treated rats, it lowered MDA to 72% of that in ALCL_3_ group.

#### Effect of cilostazol on hippocampal acetylcholinesterase (AChE) content in control and ALCL_3_-treated rats

**Fig. 6:**
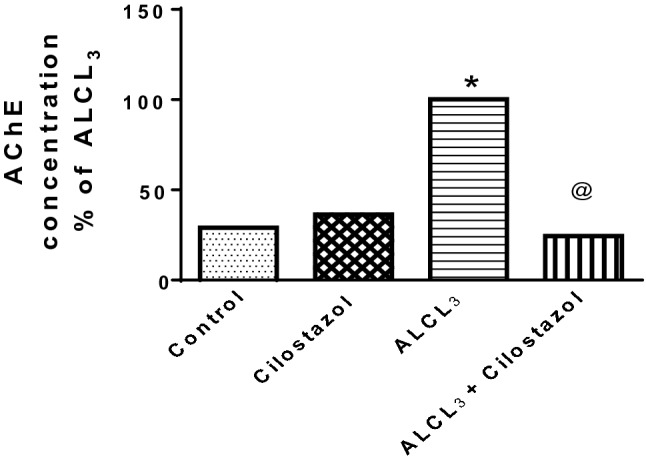
Effect of cilostazol on hippocampal acetylcholinesterase (AChE) content in control and ALCL_3_-treated rats. Values are displayed as mean ± SEM (*n* = 10). *Significantly different from control group (*p* < 0.05), ^@^significantly different from ALCL_3_ group (*p* < 0.05)

Aluminum chloride administration raised hippocampal content of AChE by 3.4-fold (Fig. 6) than when compared to control group. Administration of cilostazol alone to normal rats showed no significant effect in comparison to control group, but when given to ALCL_3_-treated rats, it lowered AChE content to 24% of that in ALCL_3_ group.

### Histopathological analysis

**Fig. 7:**
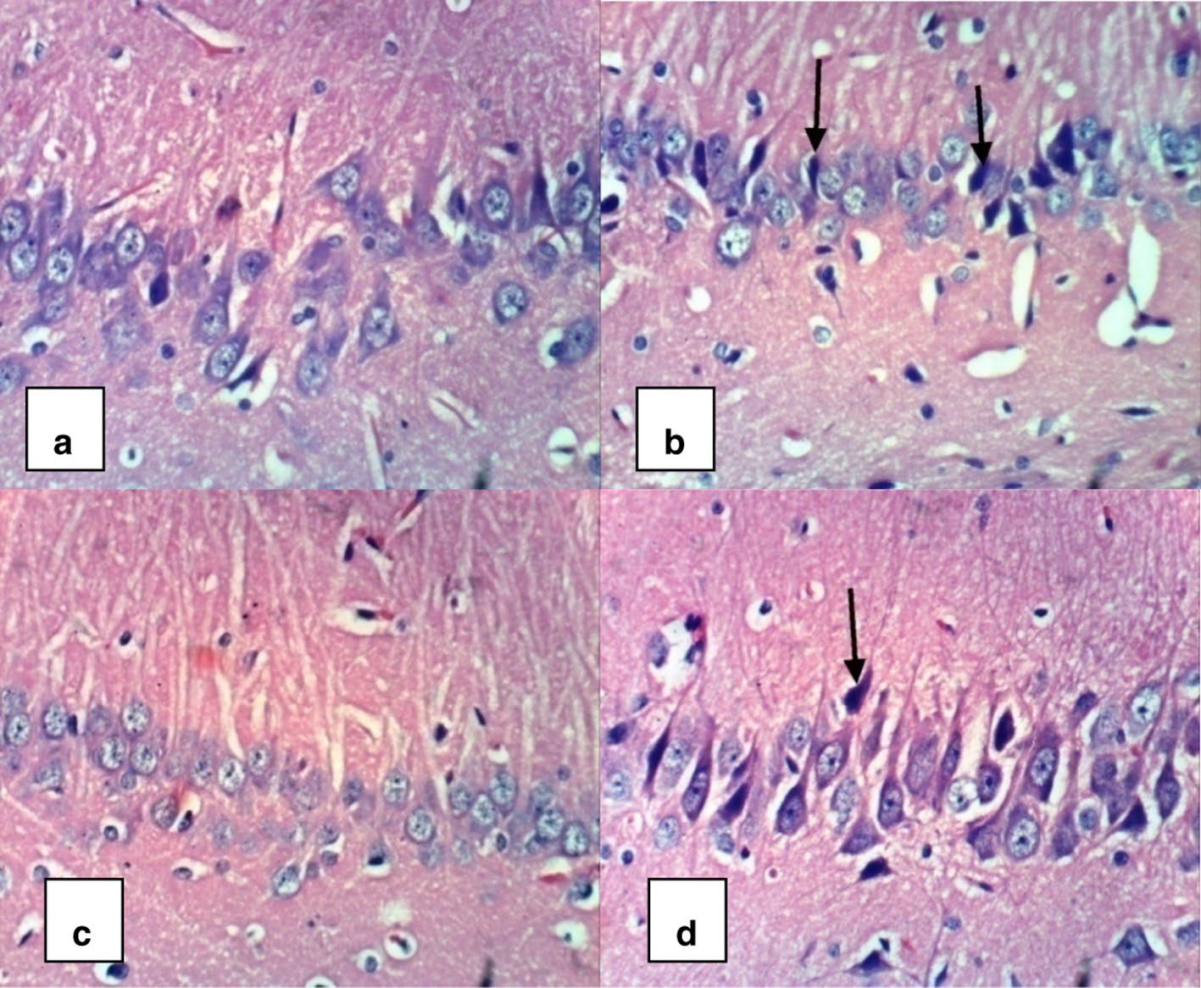
A photomicrograph of brain tissue section of rat from control and ALCL_3_-treated rats. Cilostazol alleviated the neurodegenerative pathological alterations that appeared in ALCL_3_-treated rats’ hippocampi. The above micrographical photos (H&E×400) display rats’ hippocampal sections collected on day 61 of this work. **a** Control group: revealed normal organization and structure of neuronal cells with normal appearance of the hippocampus. **b** ALCL_3_ group: revealed abnormal appearance of the neurons and their nuclei, necrosis and neurodegenerative changes. **c** Cilostazol group: showed normal appearance of the hippocampus. **d** AlCl3 + cilostazol group: showed alleviation of the neurodegenerative pathological changes that were observed in ALCL_3_-treated rats’ hippocampi

Histopathological assessment of the hippocampus also supported the biochemical findings of the present study. Microscopic examination of sections from control group revealed normal organization and structure of neuronal cells with normal appearance of the hippocampus (Fig. 7a). Sections from rats treated with ALCL_3_ revealed neurodegenerative alterations, necrosis, and abnormal appearance of the neurons and their nuclei (Fig. 7b). Cilostazol administration to normal rats revealed normal appearance of the hippocampus (Fig. 7c). Administration of cilostazol to ALCL_3_-treated rats ameliorated the observed neurodegenerative pathological changes that occurred in ALCL_3_-treated rats’ hippocampi (Fig. 7d).

## Discussion

The current study focuses on the potential protective role of cilostazol, a selective phosphodiesterase III inhibitor, on aluminum-induced memory dysfunction. Behavioral, biochemical, and histopathological alterations following aluminum chloride administration alone or with cilostazol were evaluated.

Phosphodiesterases (PDEs) are enzymes that hydrolyze phosphodiester bonds to break down cyclic guanosine monophosphate (cGMP) and/or cyclic adenosine monophosphate (cAMP). As a result, the intracellular levels of these ubiquitous second messengers are regulated. PDE inhibitors could be a powerful tool for influencing second messengers related to learning, memory, and mood (Hebb and Robertson 2007; Houslay et al. 2007).

In behavioral tests (Y-maze and Morris water maze, MWM), cilostazol was found to have a beneficial effect on memory and learning deficits caused by aluminum. Previous studies have shown that high levels of aluminum in the brain have an effect on long-term potentiation, which is thought to be the primary physiological foundation for learning and memory (Llansola et al. 1999; Shuchang et al. 2008). This study is in harmony with the other studies that showed learning and memory insufficiency following treatment with aluminum in MWM (Rani et al. 2015; Justin Thenmozhi et al. 2016; Abdel-Zaher et al. 2017) and Y-maze test (Safar et al. 2016; Alawdi et al. 2017). Other studies showed improved rats’ behavior in MWM (Watanabe et al. 2006; Lee et al. 2007; Kumar et al. 2015; Kim et al. 2016) and Y-maze (Hiramatsu et al. 2010; Maki et al. 2014) after treatment with cilostazol. PDE inhibitors have been investigated as a potential medicative intervention for cognitive disorders (Blokland et al. 2006; Reneerkens et al. 2009) via their cyclic nucleotides improving property. Cilostazol elevates cAMP in vascular cells, and has multiple impacts on the vasculature including anti-oxidation, vasodilatation, anti-inflammation, and smooth muscle cell regulation (Chen et al. 2011). The rise in cAMP activates protein kinase A, which then phosphorylates the cAMP response element-binding (CREB) protein. Phosphorylation of CREB stimulates numerous target genes, which activate new protein synthesis, thereby reinforces the existing synaptic connections and establishing new ones responsible for memory consolidation (Benito and Barco 2010). As well as, activation of CREB promotes the gene expression of neuroprotective molecules as brain-derived neurotrophic factor (BDNF) (Watanabe et al. 2006; Nishimura et al. 2007; Miyamoto et al. 2010).

In the present work, hippocampal contents of amyloid beta1-42 (Aβ_1-42_) and phosphorylated tau (p-tau) were increased together with reduced level of neprilysin (Aβ protein degrading enzyme) in aluminum chloride-treated rats. These outcomes are in accordance with Alawdi et al. (2017). Cilostazol decreased hippocampal content of Aβ_1−42_ and p-tau, while elevated neprilysin content in aluminum chloride-treated rats. Dramatic decrease in tau phosphorylation and Aβ accumulation with subsequent improvement in spatial memory and learning in Aβ_25–35_-injected mice after medication with cilostazol were observed (Tsukuda et al. 2009). Moreover, it was shown that cilostazol significantly suppressed both Aβ_1–42_ and Aβ_1–40_ aggregation and reduced Aβ production and tau phosphorylation in vitro (Lee et al. 2014; Maki et al. 2014; Schaler and Myeku 2018; Shozawa et al. 2018).

**Fig. 8:**
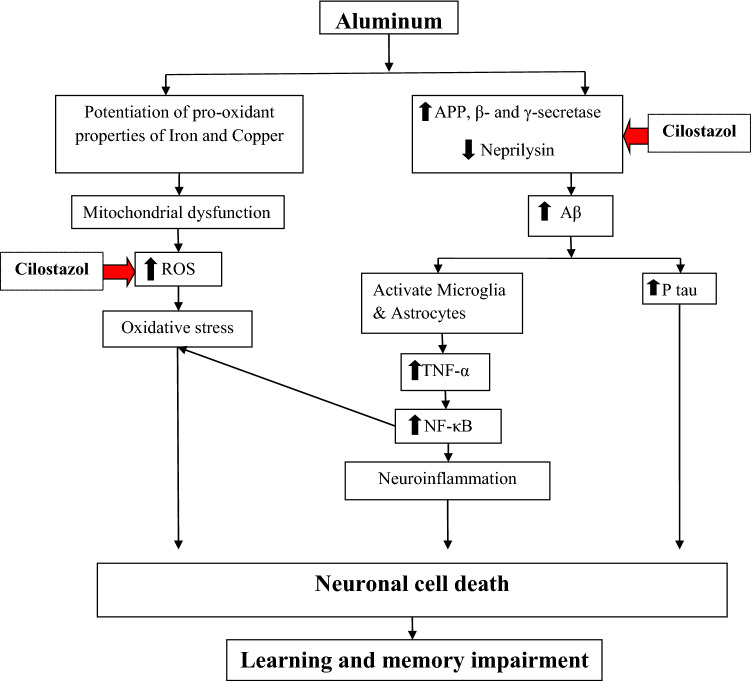
Diagram illustrating the protective actions of cilostazol against aluminum-induced memory impairment

The accumulation of Aβ protein in Alzheimer’s disease (AD) animal models results in suppression of CREB-mediated intracellular signaling pathways and impedes long-term potentiation (Puzzo et al. 2006). Cilostazol reverses this effect by the activation of protein kinase A which mediates CREB phosphorylation, and impairs the Aβ-synthesizing enzymes expression comprising β- and γ-secretase that results in decreased Aβ generation (Arendash et al. 2009). Furthermore, cilostazol-mediated cAMP might improve α-secretase activity, resulting in reduced Aβ generation (Maki et al. 2014) (Fig. 8).

Elevated hippocampal acetylcholinesterase (AChE) content was observed after aluminum chloride injection. Elevated brain AChE activity may be considered as a marker of cognitive dysfunction (Xiao et al. 2012). Other findings suggested that administration of aluminum increased AChE in mouse brain (Kaizer et al., 2005; Kumar et al., 2011). Aluminum was found to affect AChE peripheral sites and modify its secondary structure, which results in inducing its activity (Zatta et al. 1994). Cilostazol overturned the rise in AChE content in aluminum chloride-treated rats, this result demonstrated cilostazol's ability to mitigate cognitive dysfunction associated with AD. Kumar et al. (2015) detected that cilostazol significantly prevented streptozotocin-induced raise in AChE activity.

Inflammation is thought to be a key factor in AD, it triggers nervous system's defense mechanism through stimulation of microglia and astrocytes, and this ends in releasing of inflammatory cytokines and oxy radicals (Minghetti et al. 2005; Moynagh 2005). Hickman et al. (2008) noticed that the levels of insulin-degrading enzyme, neprilysin, and matrix metalloproteinase 9 (Aβ protein degrading enzymes) were dramatically decreased in older mice with the pro-inflammatory cytokines’ concomitant upregulation. FAS and FAS ligand (FAS-L) were found to be involved in Aβ protein-induced neuronal death (Millet et al. 2005). Aβ protein induces FAS-L expression via a Jun N-terminal kinase (JNK3) dependent pathway (Morishima et al. 2001).

Nuclear factor-kappa B (NF-κB) is a transcription factor that controls the production of multiple pro-inflammatory factors in inflammatory responses (Hayden and Ghosh 2004). Normally, NF-κB is binded to its biological inhibitor (inhibitor of kappa B, IκB) in the cytoplasm. IκBs are phosphorylated and then degraded by the IκB kinase (IKK) complex in response to inflammatory stimuli, resulting in free NF-κB dimmers' release (Zaky et al. 2013), displaying the DNA-binding capacity and transactivation potentials, as a result, inflammatory cytokine genes such as IL-6 and IL-8 will be expressed (Karin 2009; Brasier 2010; McFarland et al. 2013). The heterodimer of the p65 and p50 subunits is the most studied form of NF-κB that acts as a powerful gene transcription activator (Schmitz and Baeuerle 1991).

The present study demonstrated that cilostazol reduced the levels of FAS-L, tumor necrosis factor-alpha (TNF-α), and NF-κB. Cilostazol exerted anti-inflammatory activities in microglial cells and diabetic rats (Wang et al. 2008; Jung et al. 2010) by suppressing inflammatory cytokine generation and signaling (Jung et al. 2010). It also reduced raised TNF-α level and decreased the apoptosis level and cell death (Kim et al. 2002). Watanabe et al. (2006) revealed that cilostazol's neuroprotective role might be manifested via its anti-apoptotic impact through the CREB phosphorylation signaling pathway and subsequent stimulation of Bcl-2. Also, cilostazol reduced the expression of the proapoptotic protein Bax and the stimulation of apoptosis effector caspases (Oguchi et al. 2017).

Although AD pathogenesis is complex, oxidative impairment may be one of the initial events in its etiology and progression (Sultana and Butterfield 2010; Tayler et al. 2010). The hippocampal region is affected more from oxidative stress in comparison with other brain regions (Miller and O'Callaghan 2005; Yargicoglu et al. 2007). One of the most common consequences of free radical-mediated injury is lipid peroxidation, which degrades membranes directly and produces a variety of secondary products, such as aldehydes like malondialdehyde (MDA) (Slater 1984). Lipid peroxidation up-regulates β-secretase expression in vivo (Chen et al. 2008), suggesting that lipid peroxidation avoidance is a critical initial event in amyloidogenesis reduction in AD. In this study, we observed a significant amount of oxidative stress manifested itself in the form of elevated lipid peroxidation. Disruption of Golgi apparatus, reduction of synaptic vesicles, and reduced axonal mitochondrial turnover could all contribute to significant oxidative stress after aluminum administration (Bharathi et al. 2006). A raise in MDA level was noticed in rats' entire brains (Lakshmi et al. 2015), rats' hippocampi (Abdel-Zaher et al. 2017), and mice hippocampi (Jangra et al. 2015) after aluminum administration.

Cilostazol decreased MDA level when administered to aluminum chloride-treated rats. Several studies observed that cilostazol significantly reduced lipid peroxidation in the brain in different experimental models (Hiramatsu et al. 2010; Lee et al. 2010; Sahin et al. 2011; Kumar et al. 2015). PDE III inhibitors have potent anti-oxidative characteristics (Park et al. 2007; Genovese et al. 2011). Increases in intracellular cyclic nucleotides like cAMP have also been shown to reduce reactive oxygen generation, oxidative stress, and subsequent development of cellular dysfunction (Milani et al. 2005). Cilostazol inhibits oxidative stress, and thus suppresses Aβ_1–42_-induced neurotoxicity, as evidenced by decreased reactive oxygen species accumulation and elevated expression of the anti-oxidant enzyme superoxide dismutase (Oguchi et al. 2017). Moreover, cilostazol may reduce cognitive deficits by suppressing the early accumulation of lipid peroxidation products, as well as the subsequent inflammatory responses, such as the reduction of apoptotic cells (Watanabe et al. 2006).

## Conclusion

We can conclude from these findings that cilostazol reduced aluminum-induced cognitive decline through a variety of mechanisms, such as its anti-inflammatory, anti-oxidant, and anti-apoptotic properties via the cAMP/CREB phosphorylation pathway.

## Data Availability

The datasets generated during and/or analyzed during the current study are available from the corresponding author on reasonable request.
