# Correction to: Phosphodiesterase (PDE) III inhibitor, Cilostazol, improved memory impairment in aluminum chloride-treated rats: modulation of cAMP/CREB pathway

**DOI:** 10.1007/s10787-022-01034-7

**Published:** 2022-08-29

**Authors:** Mona Khalifa, Rania M. Abdelsalam, Marwa M. Safar, Hala F. Zaki

**Affiliations:** 1 Department of Clinical Pharmacy, National Cancer Institute, Cairo University, Qasr Al-Eyni Street, Fom El Khalig, Cairo, 11796 Egypt; 2 Department of Pharmacology and Toxicology, Faculty of Pharmacy, Cairo University, Qasr Al-Eyni Street, Cairo, 11562 Egypt; 3 Department of Pharmacology and Biochemistry, Faculty of Pharmacy, The British University in Egypt, Suez Desert Road, P.O. Box 43, El Sherouk City, 11837 Cairo Egypt; 4 Department of Biology, School of Pharmacy, Newgiza University, First 6th of October, Giza, 3296121 Egypt

## Correction to: Inflammopharmacology 10.1007/s10787-022-01010-1

The caption for Fig. 7 has been incorrectly placed in the Results section of original publication.

The original article has been corrected.

